# Supplementary figures and images for: Dysregulation of innate immune signaling in animal models of spinal muscular atrophy
Source: BMC Biol. 2024 Apr 25;22:94. doi: 10.1186/s12915-024-01888-z (PMC11044505; doi:10.1186/s12915-024-01888-z)

A

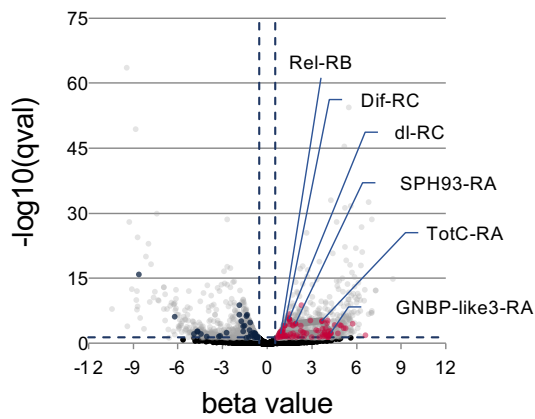

C

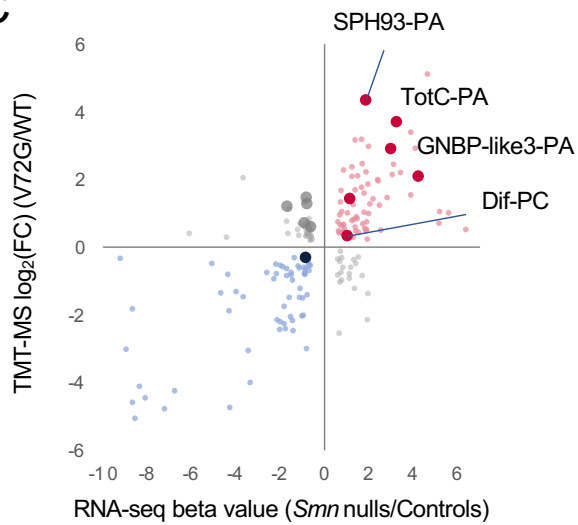

B

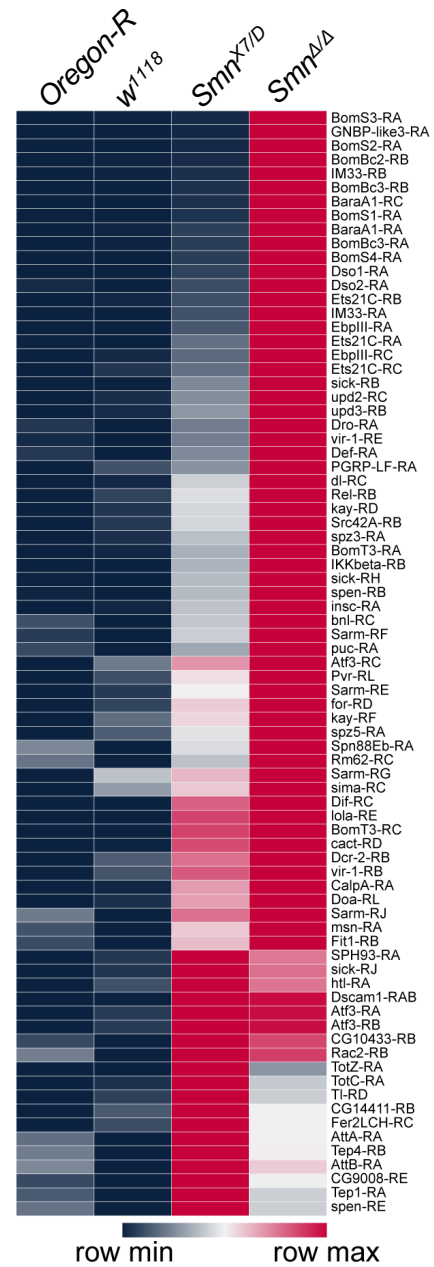

Figure S1

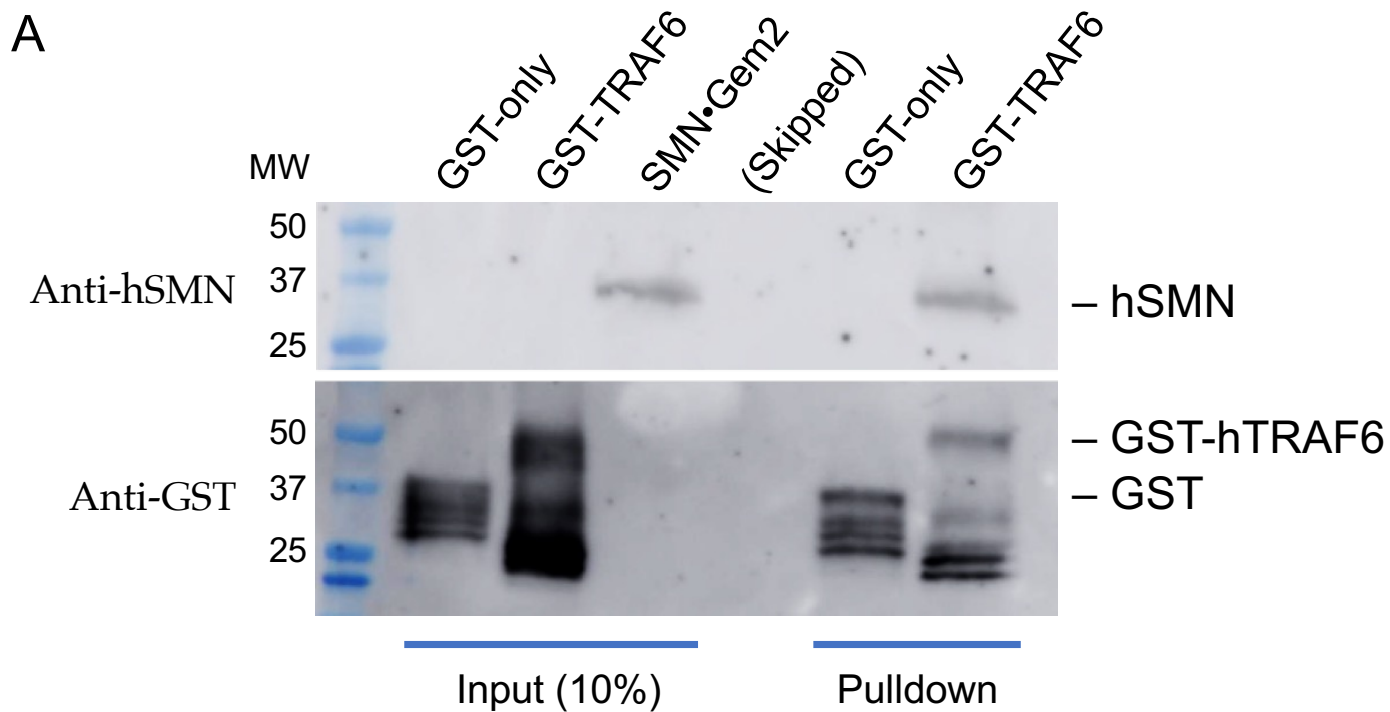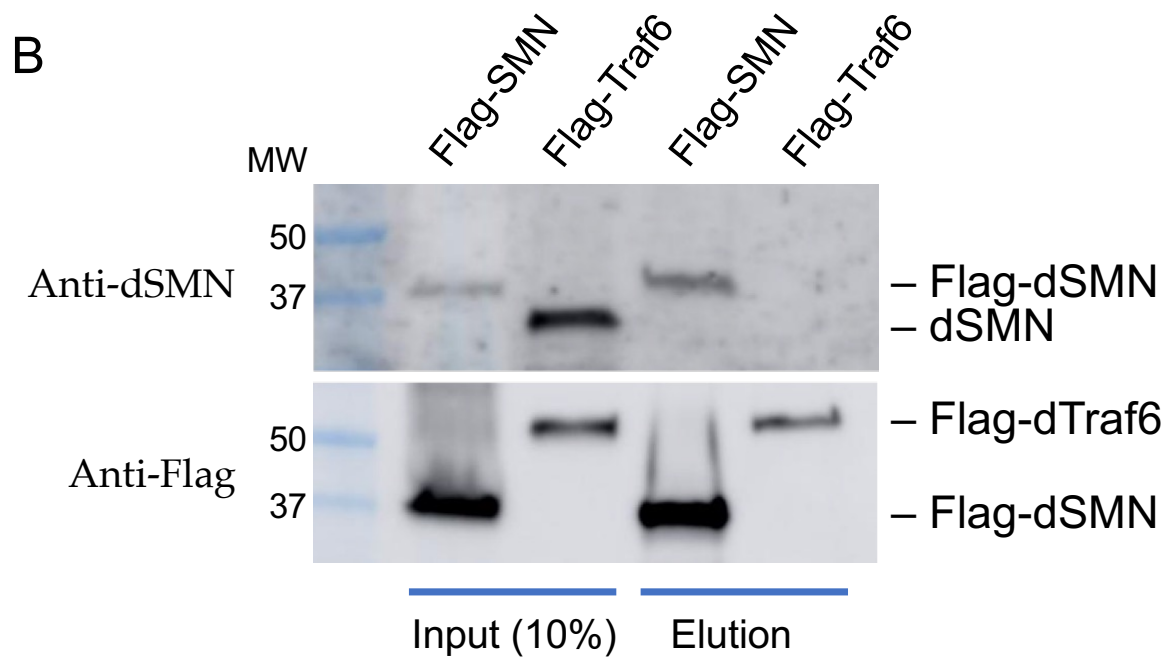

Figure S2

Supplement: Supplementary file 6 — Additional file 6: Figure S1. Isoform-specific differences in Smn mutants versus controls. A) Volcano plot of differentially expressed transcripts in Smn null animals. Transcripts associated with innate immunity are indicated with red circles and a subset of those are labeled with transcript symbols for the specific mRNA isoform difference. The axes correspond to: a Benjamini-Hochberg (False Discovery Rate (FDR) < 0.05) adjusted p-value (qval) and a Wald test-derived representation of a normalized fold change (beta factor). B) The heat map displays the respective mean transcripts per million reads for the different genotypes used in (A). The values are scaled and normalized per row (z-score). The heat map shows approximately half of the differentially expressed transcripts from (A). C) Scatter plot comparison of isoform-specific protein changes identified in the V72G proteome versus isoform-specific RNA changes found in the Smn null transcriptome. RNA and proteins associated with innate immunity are represented with larger dots and labeled. Figure S2. Evaluation of protein–protein interactions. A) GST-pulldown experiment using recombinant human SMN•Gem2 [22] and GST-TRAF6. GST and GST-TRAF6 were expressed in E.coli and purified using anti-Glutathione beads. Pulldown assays were performed and analyzed by western blotting with either anti-hSMN (top) or anti-GST (bottom) antibodies. As shown, GST-hTRAF6 interacts directly with human SMN•Gem2. B) Flag-pulldown experiment using lysates from tub-Gal4 > UAS:Flag-dTraf6 animals (Flag-Traf6) or from control animals bearing a Flag-Smn transgene [18] as the only source of SMN protein (Flag-SMN). Inputs are on the left and proteins eluted from the Flag beads following pulldowns are on the right. As shown, Flag-SMN co-purifies with itself in the control lysates but Flag-Traf6 fails to pull down endogenous dSMN in the experimental cross. [file 12915_2024_1888_MOESM6_ESM.pdf]
